# Supplementary material for: Knowledge, Compliance, and Inequities in Colon Cancer Screening in Spain: An Exploratory Study
Source: Healthcare (Basel). 2023 Sep 6;11(18):2475. doi: 10.3390/healthcare11182475 (PMC10530971; doi:10.3390/healthcare11182475)
Supplement: Supplementary file 1 [file healthcare-11-02475-s001.zip › healthcare-2527120-supplementary.pdf]

Questionnaire: Spanish CRC Screening Program.

We are conducting a survey about health issues among Spanish residents located all over the country. This is a rigorous sociological study. We would like your cooperation to answer some questions, it only takes 10 minutes to fulfill it. Thank you very much for your kindness

Before starting the survey, we like inform you that all the data you provide us is anonymous and it will be treated in accordance with current data protection law.

We guarantee that any information you provide will be processed solely for social research purposes, in a strictly confidential and anonymous manner. The data collected will only be reported in an aggregated form, so individuals cannot link to it.

QA. Please, would you please write down your age?

- Exact age (Last birthday): ________________

QB. Tell us your gender:

1. Male
2. Female

QC. In which of the following situations are you?

1. I live alone
2. I live in a residence or nursing home
3. I live in a shared apartment or campus
4. I live with a partner, without children at home
5. I live with my partner and children
6. I live without a partner, but with children at home
7. I live with other relatives (parents, siblings, etc.)
8. Other situations

QD. What is the highest level of education you have completed?

1. No studies or incomplete primary studies
2. Primary studies
3. Secondary studies
4. University studies (diploma, degree or bachelor's degree).
5. Postgraduate studies (master's or doctorate degree).

QE. Which of the following is your current main occupation?

1. Student
2. Study and work
3. Working stably
4. Working intermittently (temporary jobs, underground economy, etc.)
5. Unemployed
6. Temporary Work Incapacity (medical leave)
7. Pensioner or retiree
8. Housework (exclusively)

QF. Autonomous Community of residence

1. Andalucía
2. Aragón
3. Asturias
4. Islas Baleares
5. Canarias
6. Cantabria
7. Castilla y León
8. Castilla-La Mancha
9. Cataluña
10. C. Valenciana
11. Extremadura
12. Galicia
13. C. de Madrid
14. Murcia
15. Navarra
16. País Vasco
17. La Rioja

QG. Habitat level (Size of the municipality of residence)

1. Rural (up to 10,000 inhabitants)
2. Semi-urban (10,001 to 50,000 inhabitants)
3. Urban (50,001 to 400,000 inhabitants)
4. Large cities (More than 400,000 inhabitants)

QH. Roughly, could you indicate the net monthly income in your household, counting the contributions of all the people who live in it, including yourself?

1. Less than €1,100.
2. From €1,100 to €1,800.
3. From €1,801 to €2,700.
4. From €2,701 to €3,900.
5. More than €3,900.

QI. Could you tell us your nationality and place of birth?

1. I was born in Spain and I have Spanish nationality.
2. I was born outside of Spain but I have Spanish nationality or dual nationality.
3. I was born outside of Spain and I have the nationality of my country of origin.

QJ. In which continent is your country of birth? (Only PI.2 and PI.3)

1. Europe
2. South America
3. North America
4. Africa
5. Asia
6. Oceania

QK. In the last twelve months, would you say that your state of health has been excelent, good, fair, poor or bad?

1. Excelent
2. Good
3. Fair
4. Poor
5. Bad

QL. Would you say that the type of life you lead is very healthy, quite healthy, slightly healthy, or not healthy at all?

1. Very healthy
2. Quite healthy
3. Slightly healthy
4. Not healthy at all

Q1. How often do you usually go to the doctor for yourself?

1. Once or several times a month
2. Every two or three months
3. Every six months
4. Once a year
5. Once every few years
6. Never --> Go to Q3

Q2. What are the two main reasons why you usually go to the family doctor for yourself?

1. Specific health problems
2. Reviews or routine checks
3. For follow-up checks of a disease
4. Dispensing prescriptions
5. Administrative procedures (parts of leave, disabilities, reports, etc.)
6. Other reasons

Q3. Specifically, how often do you usually have check-ups or general medical check-ups, approximately?

1. Once a month
2. Every two or three months
3. Every six months
4. Once a year
5. Once every several years
6. Never --> Go to Q6

Q4. And is this/these medical check-up or check-ups is/are organized periodically by your employer?

1. All or mostly yes
2. Mostly are not organized by my employer
3. None is/are organized by my employer

Q5. For what reasons do you mainly carry out these tests, check-ups or medical check-ups?

1. To avoid future diseases
2. For checking-up a previous illness or disease
3. By detecting a specific discomfort
4. It is one more way to take care of my health and physical condition
5. My work company forces me, it is one more formality
6. It is a routinary process, I do it and don't think about it
7. Other reasons: ____

Q6. Taking into account your current age and state of health, would you say that, in your personal case, these types of tests, reviews or check-ups are really…?

1. Effective and necessary. *"You never know when they are going to detect a problem that it is better to catch on time".*
2. Effective, but unnecessary. *"At my age and health condition it will be very uncommon for me to detect anything, or I prefer not to know".*
3. Ineffective and unnecessary. *"They almost never detect anything important or are useless".*
4. Necessary, but ineffective. *"To be of any use, they should be deeper or more detailed".*

Q7. About this type of tests, reviews or medical check-ups, with which of these two sentences do you agree more?

1. It is not necessary to perform these tests if a person is healthy.
2. Even if a person is healthy, performing these tests is always advised.

Speaking now specifically about those tests, that are intended for the detection or prevention of cancer (in general), we will show you some sentences below. For each one, indicate your degree of agreement or disagreement.

	**Strongly agree**	**Agree**	**Disagree**	**Strongly disagree**
**Q8. A healthy lifestyle (healthy diet, adequate physical activity...) can prevent the onset of cancer**				
**Q9. Keeping a continuous follow-up with the doctor is the way to prevent or detect cancer early**				
**Q10. Carrying out specific tests on certain types of cancer is the best way for its early detection and treatment**				
**Q11. The onset of cancer is more a matter of chance or genetics, and there is little we can do to prevent it.**				

Q12. Let's keep talking about cancer. You or an immediate family member (father/mother, partner/spouse, child) has suffered or is suffering from cancer.

Personally **Inmediate Family member**

1. Yes
2. No

(If the answer is affirmative, on a personal or family level, go to Q13. If the answer is negative in both cases, go to Q15).

Q13. Please indicate the type or types of cancer that you or your close family member(s) (father/mother, partner/spouse, child) have or have had

(* Possible multiple choice)

1. Cervix.
2. Colon and rectum (Colorectal).
3. Stomach.
4. Larynx / trachea.
5. Leukemia.
6. Lymphoma.
7. Breast
8. Melanoma.
9. Mesothelioma.
10. Myeloma.
11. Ovary.
12. Pancreas.
13. Prostate.
14. Lung.
15. Kidney.
16. Uterus.
17. Bladder.
18. Others.

Q14. Below this text you will see some types of cancer. From what you know about cancer or what you have heard, which ones would you say there are the three most frequently diagnosed types nowadays in Spain?

The most frequent **2nd most frequent** **3rd most frequent**

1. Colon and rectum (colorectal)
2. Lung
3. Breast
4. Prostate
5. Bladder
6. Pancreas
7. Uterus
8. Stomach
9. Leukemia (blood cancer)

Now we would like to focus exclusively on colon (or colorectal) cancer

Q15. Before this survey, had you heard of this type of cancer?

1. Yes
2. No

Q16. What would you say is your personal risk of being diagnosed with colon (or colorectal) cancer in your lifetime?

1. Very high
2. High
3. Low
4. Very low

Q17A. [Only very high or high.] For what reasons do you think that? (select maximum three)

1. I have a family history of cancer
2. I have ever noticed something that could be a symptom of colorectal cancer
3. I lead a sedentary life or do little physical exercise
4. I have an unhealthy diet
5. I am overweight or obese
6. I am a smoker
7. I drink alcohol regularly
8. I am exposed to toxic substances at work
9. Due to air pollution/cars
10. I suffer from too much stress
11. I think it can happen to anyone
12. I begin to have an age where it is usual to be diagnosed
13. The incidence of this type of cancer is increasing among the population
14. Others. Specify:

Q17B. [Only low or very low.] For what reason? (select maximum three)

1. I have no family history of cancer
2. I have never had any symptoms
3. Because I have check-ups with a certain periodicity
4. I have an active life, practising physical exercise
5. I follow a healthy and balanced diet
6. I have a good weight and physical condition
7. I don't smoke
8. I don't drink alcohol or only occasionally
9. I'm still young for that
10. I'm too old for that
11. I am not exposed to toxic substances
12. It is very unlikely that it will happen to me
13. I prefer not to think about it
14. Others. Specify:

Q18. From what you know or have heard, would you say that, among the different types of cancer, the rate of occurrence of colorectal cancer in the general population is...?

1. Very high (one of the most frequently occurring types of cancer)
2. High
3. Low
4. Very low (one of the less frequently occurring types of cancer)
5. Don't know

Q19. And do you think colorectal cancer affects men or women more?

1. Much more to men
2. Something more to men
3. Both genders equally
4. Something more to women
5. Much more to women
6. Don't know

Q20. In terms of age, which people would you say are most affected by colorectal cancer?

1. Above 70 years
2. Between 50 and 70 years
3. Between 30 and 50 years
4. Under 30 years
5. At all ages equally
6. Don't know

Q21. Regarding the mortality rates (or survival) of colorectal cancer, what would you say it is…?

1. One of the most dangerous, even if detected early
2. It is dangerous, but only if it is not detected in time
3. It is relatively easy to treat and cure if caught early
4. It is one of the easiest to treat and cure even without early detection
5. Don't know

Q22. Do you know or have you ever heard about the colon/colorectal cancer early detection (or screening) program?

1. Yes
2. No, I don't think I've ever heard of it.

Q23. This program includes a Fecal Occult Blood Test (FOBT). Have you heard of this particular test prior to this survey?

1. Yes
2. No, I don't think I've ever heard of it.

Q24. This program for the early detection of colorectal cancer is promoted and managed by the health authorities. To be confirmed, have you ever received a letter from the Department of Health of your region inviting you to personally participate in this detection program?

1. Yes, I have received a letter about it
2. No, I have never received a letter about it --> Go to Q26

Q25. Let's talk about the letter inviting you to participate in this program (if you have been sent more than one letter, think about the first time you received it)

Q25.1 Were you surprised to receive the letter?

1. Nothing surprised
2. I was a bit surprised
3. I was quite surprised

4. I was very surprised
5. I don't remember

Q25.2 What was your reaction when you received it?

1. Very positive, I thought that this type of initiative is important and necessary --> Go to Q25.3
2. More positive than negative, although it has its negative parts I thought that this type of tests are generally beneficial --> Go to Q25.3
3. More negative than positive, although it has its good parts I thought the drawbacks were too many --> Go to Q25.4
4. Very negative, I thought that these types of initiatives are really useless --> Go to Q25.4
5. I don't remember --> Go to Q26

Q25.3 Which ones of the following reasons or aspects were the two main ones that, at the time of receiving the letter, led you to value it positively?

1. The information in the letter was clear and simple
2. I understood the benefits of taking the test
3. I easily understood the process of carrying out the test
4. From what it explained, the test was easy for me to pick up and deliver
5. From what it explained, the test was easy and comfortable for me to do
6. The fact that the test was free of charge
7. I think that being able to participate in this test is important
8. Other. Specify:

(Go to Q26)

Q25.4 which ones of the following reasons or aspects were the two main ones that, at the time of receiving the letter, led you to value it negatively?

1. I did not expect the invitation, it was strange for me to receive it
2. I thought it was not for me to take the test due to age, I am still young
3. I was scared to receive the invitation
4. The information on the letter was unclear
5. The tone of the letter was somewhat aggressive, negative
6. I think that the test they explained is unpleasant to perform
7. There was little explanation about the benefits of getting tested
8. There was little explanation about the process of taking the test
9. From what they said, taking the test was complex, I did not understand what I had to do
10. I found the delivery process difficult or cumbersome after completing it
11. The process of going to pick it up was complicated for me
12. I don't like this kind of tests
13. Other. Specify:

Q26. Whether you have received a letter from the Health Department of region for the early detection of colorectal cancer or not , have you ever undergone this test?

1. Yes --> Go to Q27
2. No --> Go to Q28

Q27. Which ones of the following reasons or aspects, were the three main reasons why you participated in this test?

Most important reason Second most important reason Third most important reason

1. It was simple and comfortable to perform
2. It was easy to submit and receive the results
3. By age it was the right time
4. A family member/friend has or had this cancer
5. I believe that if this cancer is detected early, it can be cured in most cases
6. I thought I might have symptoms of the disease
7. For ruling out the possibility of having the disease
8. It was recommended to me by a family member or friend
9. My doctor recommended it to me
10. My pharmacist recommended it to me
11. People close to me told me about a positive experience with the test
12. Other. Specify:

Q27.1 Before you received this letter, had you heard about this test from anyone? Indicate, if applicable, who had told you about it [MULTIPLE CHOICE]

14. A close relative
15. A friend, neighbor, or co-worker
16. My family doctor
17. A specialist doctor (at any hospital visit, outside of primary care)
18. A pharmacist
19. Other. Specify:

(Go to Q31)

Q28. Which ones of the following reasons or aspects were the three main reasons why you did not participate in this test?

more important reason second most important reason Third most important reason

1. I forgot / due to carelessness
2. I did not receive the letter/notification
3. I didn't have time to pick it up or deliver it
4. I'm still too young for it, it can be done later
5. I don't usually use public healthcare, I prefer private healthcare
6. There is a saturation of public health
7. Due to COVID, I preferred to stay away from medical centers
8. I had no symptoms and I was not in poor health
9. I was a little scared of the result
10. I found the type of test uncomfortable or embarrassing (stool sample)
11. I found it unpleasant to do
12. People close to me had a negative experience with this test
13. I had previously had a test to detect this cancer
14. Other. Specify:

Q29. And if you received an invitation from your Regional Health Department to personally participate in this program for the early detection of colon/colorectal cancer, which includes a Fecal Occult Blood Test, would you perform this test?

1. Yes, for sure--> Go to Q30.1
2. Probably yes--> Go to Q30.1
3. Probably not--> Go to Q30.2
4. No, surely--> Go to Q30.2
5. I don't know--> Go to Q31

Q30.1. Which ones of the following reasons would be the three main reasons why you would participate in this test?

1. For ruling out the possibility of having the disease
2. If this cancer is detected early, it can be cured in most cases
3. By age it would be the right time
4. A relative/friend has or has had this cancer
5. I think I may have symptoms of the disease
6. Others

(Go to Q31)

Q30.2. And what would be the three main reasons why you would not participate in this test?

1. I have no symptoms and I am not in poor health
2. There is a saturation of public health
3. I find the type of test uncomfortable or embarrassing (stool sample)
4. I find it unpleasant to do
5. Due to COVID, I prefer to stay away from medical centers
6. The result scares me a little
7. I don't usually use public healthcare, I prefer private healthcare
8. I'm still too young for it, it can be done later
9. Others

(Go to Q32)

Q31. Related to this, how much do you agree or disagree with the following statements?

	**Strongly agree**	**Agree**	**Disagree**	**Strongly disagree**
**Q31.1 Carrying out this Fecal Occult Blood Test (FOBT) is not as necessary when the person does not present symptoms and is in good health**				
**Q31.2 This type of test should be aimed at older people**				
**Q31.3 With this type of test you cannot be sure of the results**				
**Q31.4 Performing this test does not ensure the possible occurrence of colon cancer in the future**				
**Q31.5 It is an uncomfortable test, unpleasant to perform**				

**Q31.6 Uncertainty about the result of this type of test can be distressing**				

(Go to Q33)

Q32. Related to this, how much do you agree or disagree with the following statements?

	**Strongly agree**	**Agree**	**Disagree**	**Strongly disagree**
**Q32.1 Really, performing this Fecal Occult Blood Test (FOBT) is quite simple to perform.**				
**Q32.2 The benefits of performing this test far outweigh the drawbacks of performing it**				
**Q32.3 When it comes to cancer, its early detection is always fundamental for its subsequent treatment**				
**Q32.4 Performing this type of test is one more way to take care of your own health**				

(To all)

Q33. To conclude would you say that, in general, is the information that the population receives about colon cancer and its prevention should be further or is it already broad enough?

1. I think there is very little or no information about this prevention program
2. I think that the information that exists about this program is less than necessary
3. I think that the information on this program is sufficient
4. I think that the information on this program is more than adequate
5. Don't know

Q34. In general, what is your opinion about the program for the early detection of colorectal cancer that we have talked about in the survey?

1. Very positive
2. Positive
3. Negative
4. Very negative
5. Don't know

Q35. Finally. Could you tell us what type of health insurance (public or private) you have?

1. Public Health (Social Security)
2. State mutual funds (MUFACE, ISFAS, MUGEJU, etc.) covered by Social Security
3. State mutual funds (MUFACE, ISFAS, MUGEJU) covered by private insurance
4. Private medical insurance, arranged individually (medical societies, Professional Associations, etc.)
5. Medical insurance arranged by the company
6. I don't have health insurance

End of the survey.

Thank you very much for your help
